# Correction: Comparative study of thoracolumbar fascia changes in weightlifters with and without low back pain

**DOI:** 10.1371/journal.pone.0360981

**Published:** 2026-10-16

**Authors:** Emma Feng Ming Zhou, Carmelo Pirri, Xiaoxiao Zhao, Juhua Peng, Tao Wen, Jianhui Fang, Chufeng Zhou, Carla Stecco, Siu Ngor Fu

The eighth and ninth authors are listed out of order. The correct order is: Emma Feng Ming Zhou^1^, Carmelo Pirri^2^, Xiaoxiao Zhao^2^, Juhua Peng^3^, Tao Wen^3^, Jianhui Fang^3^, Chufeng Zhou^3^, Carla Stecco^2^, Siu Ngor Fu^1^

**1** Department of Rehabilitation Sciences, The Hong Kong Polytechnic University, Hung Hom, Hong Kong, **2** Department of Neurosciences, Institute of Human Anatomy, University of Padova, Padova, Italy, **3** Department of Rehabilitation, Guangdong Sports Hospital, Guangzhou, Guangdong, China

The ninth author, Siu Ngor Fu, is incorrectly noted as the corresponding author. The correct corresponding author is Carla Stecco. Carla Stecco’s email address is: carla.stecco@unipd.it.
