# Supplementary material for: Comparative study of thoracolumbar fascia changes in weightlifters with and without low back pain
Source: PLoS One. 2025 Dec 19;20(12):e0338718. doi: 10.1371/journal.pone.0338718 (PMC12716693; doi:10.1371/journal.pone.0338718)
Supplement: S1 Table — (DOCX) [file pone.0338718.s001.docx]

**Supplementary Table 1 Intra-rater reliability of TLF measurements using ultrasound**

|  | **ICC_3,1_**  **(95%CI)** | **Mean (SD) (kPa)** | | **SEM (kPa)** | | **MDC (kPa)** | |
| --- | --- | --- | --- | --- | --- | --- | --- |
|  |  | **Test 1** | **Test 2** | **Test 1** | **Test 2** | **Test 1** | **Test 2** |
| **Right thickness** | 0.984 (0.96, 0.99)** | 1.56 ± 0.43 | 1.52 ± 0.42 | 0.05 | 0.05 | 0.15 | 0.15 |
| **Left thickness** | 0.978 (0.78, 0.95)** | 1.51 ± 0.35 | 1.49 ± 0.39 | 0.05 | 0.06 | 0.14 | 0.15 |
| **Right stiffness** | 0.923 (0.79, 0.97)** | 46.29 ± 15.59 | 48.41 ± 14.28 | 4.33 | 3.96 | 11.99 | 10.98 |
| **Left stiffness** | 0.861 (0.64, 0.95)* | 48.37 ± 11.97 | 47.04 ± 12.09 | 4.46 | 4.51 | 12.37 | 12.49 |

Abbreviations: CI = confidence interval; ICC = intra-class correlation; MDC = minimum detectable change; SD = standard deviation; SEM = standard error of measurement; TLF = thoracolumbar fascia. *: good reliability: ICCs = 0.75–0.9; **: excellent reliability: ICC > 0.9
